# Transcriptome analyses reveal new insights on key determinants of perineural invasion in high-grade serous ovarian cancer

**DOI:** 10.3389/fcell.2023.1109710

**Published:** 2023-09-20

**Authors:** Zhen Zheng, Xiao Li, Guoqiang Chen, Jing Chen, Xiaolu Zhu, Yincheng Teng

**Affiliations:** ^1^ Department of Obstetrics and Gynecology, Shanghai Sixth People’s Hospital Affiliated to Shanghai Jiao Tong University School of Medicine, Shanghai, China; ^2^ Department of Obstetrics and Gynecology, Changzheng Hospital, Naval Medical University, Shanghai, China

**Keywords:** ovarian cancer, neural invasion, TAS2R receptor family proteins, tumor immune microenvironment, transcriptome

## Abstract

Perineural invasion (PNI) is a pathological feature of many cancers associated with poor outcomes, metastases, and recurrence. In relation to ovarian cancer (OC), there is no information about PNI’s role and mechanisms. Our study found that patients with PNI-positive symptoms had significantly shorter overall survival (OS) time than patients with PNI-negative symptoms. Multivariate analyses demonstrated that PNI represented a substantial independent prognostic factor in OC patients. At the transcriptome level, it is noteworthy that PNI positivity was negatively correlated with the degree of infiltration of immune killer cells in OC tumor tissues, including macrophage, central memory CD4 T-cell, natural killer cells, monocyte, and central memory CD4 T-cell. The results of this study revealed that TAS2Rs proteins were markedly upregulated in PNI-positive OC tissues and predicted poor prognoses. Moreover, Immunohistochemical analysis demonstrated that the TAS2R10 protein was associated with poor prognoses and PNI in OC. Consequently, we found for the first time that PNI was a powerful predictor of poor prognosis in OC and analyzed its expression pattern and some preliminary biochemical characterization, providing new clues for guiding clinical prevention and treatment of OC.

## 1 Introduction

The nervous system plays a crucial role in the invasion of cancer. Neurotropic carcinomatous invasion (PNI) occurs when tumors invade and spread along nerve sheaths, which can prove to be one of the more metastatic routes (Amit et al., 2016). There is widespread recognition that PNI is a significantly poor prognosis signature in all solid tumors (Entschladen et al., 2006; Deborde et al., 2016). The most probable mechanism of PNI is that cancer cells secret neurotrophic factors that attract nerve fibers and stimulate nerve growth, which can promote cancer growth (Bapat et al., 2011). Unfortunately, researchers are still in the early stages of understanding how PNI is caused by complex signaling between nerves, cancer cells, and other molecules (Cervantes-Villagrana et al., 2020). Consequently, the underlying interplay between cancer cells and nerves within the tumor microenvironment is crucial (Hu et al., 2022; Li et al., 2022).

Most cases of OC are the deadliest gynecological malignancy, which is discovered when they are advanced (Menon et al., 2021). Recent studies have revealed the presence of innervation in OC (Tapia et al., 2011; Retamales-Ortega et al., 2017). Neurotrophic factors, a family of growth factors, are highly expressed in OC, promote the growth, metastasis and aggression of cancer cells, and are strongly associated with adverse outcome (Amit et al., 2016; Retamales-Ortega and Orostica, 2017). Neurotrophic factors contain five kinds of proteins including NGF, BDNF, and NT3-6. Related studies have found that neurotrophic factors secreted by OC cells, including BDNF and NGF, interact with receptors on nerve cells to add innervation in tumors (Szubert et al., 2016; Retamales-Ortega and Orostica, 2017). However, research on the role of PNI in OC is lacking.

In this study, we explored for the first time the molecular characteristics of PNI in OC. H&E staining was used to assess PNI and diagnostic images of 73 ovarian plasmacytomas obtained from TCGA were arranged into PNI-positive and PNI-negative groups for subsequent analysis. This is the first study to describe the genomic, transcriptomic, and immunological spectrum analyses of PNI molecules in OC. Consequently, we identified PNI as an independent predictor of OS in OC patients. The bitter taste sensing type 2 receptors (TAS2Rs) played an important role in the invasion of the perineural space in OC, providing new clues for guiding clinical prevention and treatment of OC.

## 2 Materials and methods

### 2.1 Histopathology image

Histopathological images of 106 cases of high-grade serous OC were derived of the TCGA data base. Publicly available histopathological images were used for the research. We got informed consent from TCGA data. From the initial dataset, 73 samples with relevant phenotypes were included for further analysis. The remaining 33 samples were excluded due to the absence of pertinent phenotypic information.

### 2.2 Data acquisition

Data of OC survival (n = 731), phenotype (n = 758), and gene expression RNAseq (n = 379) were obtained from the GDC TCGA OV cohort (https://xena.ucsc.edu/). OC somatic mutation data (n = 418) were obtained in the TCGA GDC portal (https://portal.gdc.cancer.gov/).

### 2.3 Evaluation of perineural involvement

Perineural invasion (PNI) was defined as tumor cells covering more than one-third of the length of the nerve. The two pathologists independently repeated the radiographs and divided them into two groups: PNI-positive images of OC cells covering the nerve and negative images of OC cells not covering the nerve. At the same time, the nerve diameter together with the distance between OC cells and nerves were measured and counted.

### 2.4 Differential expression analysis

We made an intersection of 106 samples containing OC sections and clinical data as well as gene expression RNA-seq to obtain 73 samples. After reviewing the whole slides, two pathologists divided the samples into 14 PNI-positive cases and 59 PNI-negative cases for differential expression analysis. Differential expressed genes (DEGs) were examined among the positive PNI samples and the negative PNI samples using the limma package. A |log2FC|>1 and a *p*-value of 0.05 were used as the threshold for DEG.

### 2.5 Mutation analysis

An analysis of the TCGA database was conducted to determine whether the mutation frequency differed between PNI-positive samples and PNI-negative samples. Using R software, mutation data and gene mutation landscapes were analyzed and plotted using the ComplexHeatmap package [version 2.2.0 (15)].

### 2.6 Immune cell infiltration

Using a previously developed and validated computational technique, we determined the degree of immune cell infiltration in OC. The Tumor Immune Estimation Resource (TIMER) database was used to obtain data on immune cell infiltration. A quantitative analysis of the level of 24 kinds of immune cells infiltrating in each sample and an evaluation of the effect of genes on immune infiltration were conducted using ssGSEA.

### 2.7 Pathway enrichment analysis

DEGs were explored using GO enrichment and KEGG pathway assays to discover underlying biologic process, cell composition, and the functional molecules. A gene set for each gene set was retrieved from the Molecular Signatures Database (MsigDB) on the basis of these two gene sets: ‘hallmark (h.all) and c2. cp.KEGG.v6.2. symbols’. Using the Benjamini–Hochberg procedure, *p*-values were adjusted to allow for multiple testing of many genes, which results in calculating the false discovery rate (FDR).

### 2.8 Prognostic analysis

The Kaplan-Meier method with log-rank tests was used to analyze clinical correlations, and the *2 test was used for survival analysis. In order to calculate the hazard ratios (HR), R software and the “survival” R package were used for the Cox proportional hazards analysis. In addition, four gene signatures in the TAS2R family have been identified through multivariable analysis of Cox regression as an independently prognostic agent. LASSO (Least Absolute Shrinkage and Selection Operator) was used to minimize this risk. The prognostic analysis of TAS2R3, TAS2R10, and TAS2R50 in Figure 5D was conducted using an online analysis tool (http://kmplot.com/analysis/).

### 2.9 Histological analysis

Ethical approval was obtained from the institutional ethics committee of shanghai sixth people’s hospital for the study protocol. The study aimed to investigate the expression of TAS2R10 in PNI negative and positive ovarian cancer. For this purpose, we used a total of 100 samples of ovarian cancer that were collected between June 2012 and December 2021. A section of paraffin-embedded tissues was stained with HE and immunohistochemically examined. A primary antibody was incubated at 4 C overnight with sections for immunohistochemistry analysis (Hu et al., 2021). A secondary antibody was incubated afterward. We purchased TAS2R10 (A15156, 1:100) primary antibody from Abclonal in China.

### 2.10 Immunofluorescence staining

In the same way as previously described (Jiang et al., 2019), immunofluorescence staining was performed in the present study. Immunofluorescence was carried out using the following primary antibodies: S100 (90393S, 1:100) and GFAP (3655S. 1:100) were obtained from CST.

### 2.11 Statistical analysis

In order to analyze somatic variants efficiently and comprehensively in OC, we used the R/Bioconductor package Maftools. Kruskal–Wallis tests and ANOVAs were performed as statistical tests for three groups. Tests of difference between the two groups were conducted using the Wilcoxon rank sum test. Spearman correlation tests were used for correlation analyses. Calculations of hazard ratios (HRs) and 95% confidence intervals (Cis) were performed using Cox proportional hazard regression. Multivariate analysis of prognosis was performed using the multivariate Cox regression model. Assuming that all statistical tests were 2-sided, a *p*-value of < 0.05 was defined as statistically significant. In this study, R software (version 4.0.2) was used to conduct statistical analysis.

## 3 Results

The workflow of this study was as shown in Supplementary Figure S1.

### 3.1 Integrated molecular characterization of PNI in OC

**FIGURE 1:**
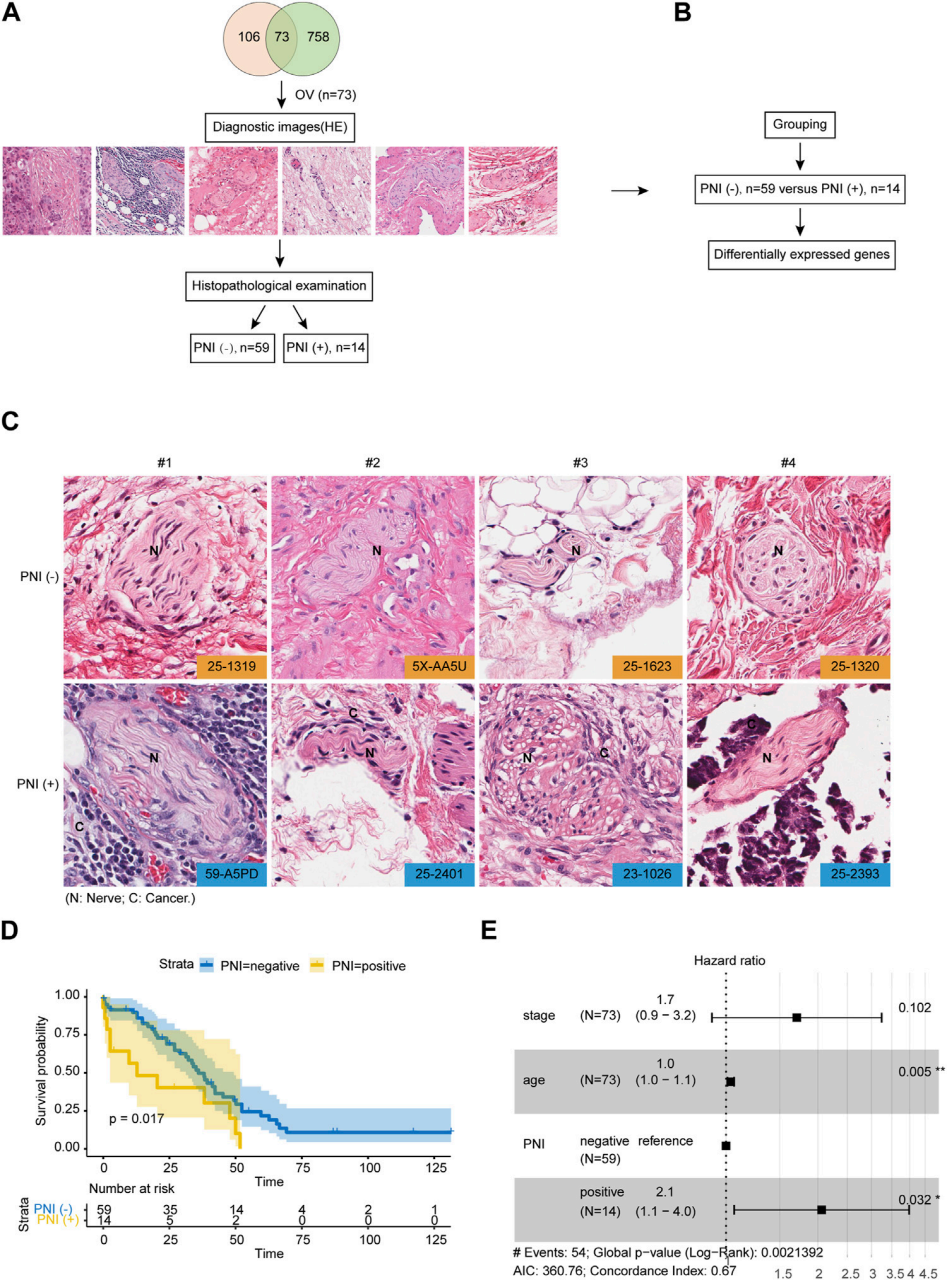
Integrated Molecular Characterization of PNI in OC. **(A)** OC samples (n = 73) in the TCGA cohort with the diagnostic image were subjected to histopathological examination for defining PNI status. **(B)** Samples were divided into two groups: PNI-positive patients (n = 14), and PNI-negative patients (n = 59) for further analysis. **(C)** Representative PNI images of PNI-positive and PNI-negative groups. **(D)** Kaplan-Meier overall survival curve showing the prognostic value of PNI status of OC patients from TCGA cohort (log-rank test). **(E)** Multivariate analysis of prognostic parameters for OC patient survival in TCGA cohort.

A total of 106 hematoxylin and eosin (H&E) stained histopathological images of full sections of serous ovarian cancer were acquired in The Cancer Genome Atlas (TCGA), and the accompanying pathology report. The downloaded OC clinical TCGA data included phenotypic information for 758 samples, and 73 samples had information on both gene expression profiles, effective survival, and histopathology images. All histopathology images of 73 patients had been assessed by two independent pathologists to define the PNI status. By stratifying the patients according to the status of PNI, we divided them into two groups: PNI-positive patients (n = 14), and PNI-negative patients (n = 59) for further analysis (Figures 1A,B). Figure 1C shows representative images of OC samples with positive or negative PNI. Among patients with positive PNI, the cumulative survival rate was lower than that of patients with negative PNI (Figure 1D). Our study revealed that there were no significant differences between the two subtypes of ovarian cancer in terms of clinical stage, as assessed by 73 patients. However, multivariate analysis showed that PNI positivity was a critical predictor of worsening survival (HR = 2.1, *p* = 0.032), as depicted in Figure 1E. These findings suggest that the prognostic value of PNI positivity is independent of the stage of the disease, underscoring the potential usefulness of PNI as a valuable prognostic marker for ovarian cancer patients. The identification of PNI positivity as a prognostic marker could inform the development of new therapeutic strategies aimed at improving patient outcomes, particularly for those with advanced-stage disease. These results have important implications for the management of ovarian cancer and warrant further investigation.

### 3.2 Difference of genomic landscape related to PNI status

**FIGURE 2:**
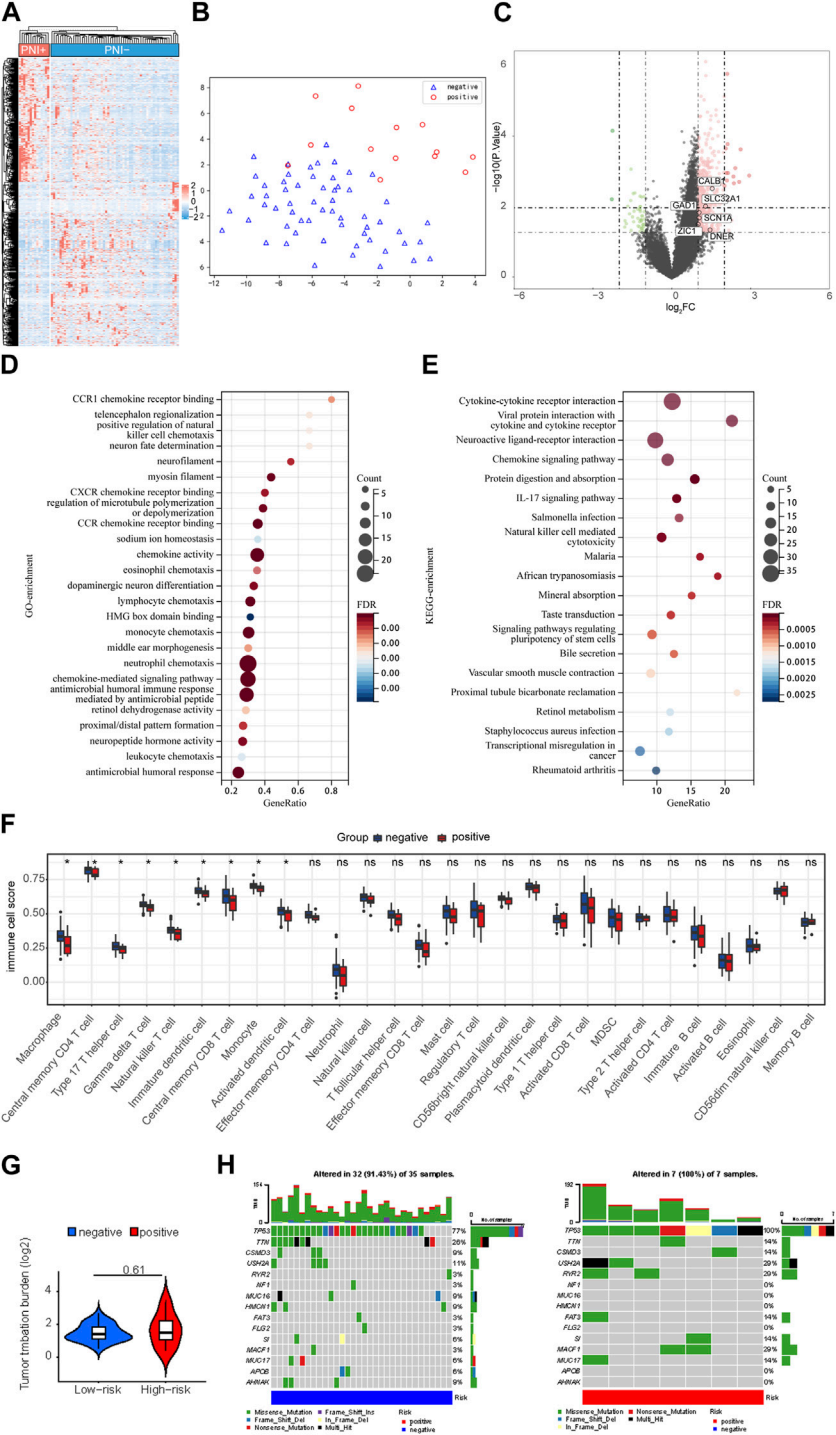
Difference of genomic landscape Related to PNI Status. **(A)** Horizontal cluster of genes with differentiated expression among PNI-positive and PNI-negative samples. Red represents upregulation; blue represents downregulation. Each row denotes a sample; each line denotes a differentially expressed gene. **(B)** t-SNE analysis for PNI-positive and PNI-negative groups revealed two disjoint populations. Blue, PNI-negative samples; Red, PNI-positive samples. **(C)** The volcano map structured with *p* < 0.05 and |logFC|≥1 as cutoff criteria. Red, upregulated genes; blue, downregulated genes. **(D)** Gene ontology feature rich analysis of difference expression genes. **(E)** Demonstrate the KEGG enrollment analysis of a difference expressed gene. All displayed enrichment routes are remarkably differential. **(F)** Comparative 24 immune cell subtypes among PNI-positive and PNI-negative cohorts. **(G)** The tumor mutation burden (TMB) within the PNI-positive and PNI-negative groups. **(H)** Comparison of the frequently mutated genes among PNI-positive and PNI-negative cohorts.

According to the TCGA-OC dataset, 2,805 genes were differentially expressed in the PNI-positive *versus* PNI-negative samples (Figure 2C). Using the following criteria, differentially expressed genes were defined as fold change ≥2 or ≤0.5 and FDR ≤0.01. The hierarchical clustering of gene expression level data revealed a different landscape between PNI-positive and PNI-negative groups (Figure 2A). In this context, t-SNE (Van der Maaten et al., 2008; Van Der Maaten, 2014) was used to compare the two groups being studied, and the resulting analysis clearly showed a distinction between the two groups, as demonstrated in Figure 2B. This finding suggests that the groups may have different underlying characteristics, which could have important implications for further research and potential clinical applications (Figure 2B). We have identified several gene expression markers for neurons, including CALB1, SLC32A1, SCN1A, ZIC1, DLGAP1, CPNE6, DNER, and GAD1, that are significantly upregulated in PNI + samples (Figure 2C). These findings suggest that neuronal signaling and communication may play a critical role in the perineural invasion of ovarian cancer. The upregulation of these markers provides insight into the potential mechanisms underlying the occurrence of ovarian cancer perineural invasion. To gain further insight into the types of nerves invaded by ovarian cancer cells, we used the panglaodb database to identify gene signatures for nerves and intersected these signatures with the upregulated DEGs in our study. Our analysis revealed that the expression of HTR2C, a characteristic marker gene for parasympathetic neurons, was significantly upregulated in PNI of OC (Supplementary Figure S2A and Supplementary Table S1). These findings suggest that the nerve fibers invaded by ovarian cancer cells may be parasympathetic in nature. In order to discover the potential biological functions and signaling pathways of PNI, we obtained GO and KEGG annotations of the 2,805 DEGs in OC based on functional enrichment analysis. According to GO enrichment analysis, these genes play a critical role in multiple Neural signal-related pathways including neuropeptide hormone activity, dopaminergic neuron differentiation, neuron fate determination, and neurofilament (Figure 2D). Multiple pieces of evidence demonstrated the chemokines and their receptors are responsible for incusing cancer cells to invasion (Erin et al., 2022). According to previous studies, CX3CL1 and its unique CX3CR1 receptor are involved in the progress of PNI in pancreatic cancer cells (Hirth et al., 2020; Huang et al., 2020). Likewise, we found that these genes also were strongly associated with chemokine receptors pathways including CCR1, CXCR, and CCR chemokine receptors binding in OC (Figure 2D). KEGG enrichment analysis found the pathways involved in the regulation of neuronal activity, such as the neuroactive ligand-receptor interaction pathway and the chemokine signaling pathway (Figure 2E). These findings suggest that multiple signaling pathways, including those related to neural signaling and chemokine signaling, may be involved in the pathogenesis of PNI in ovarian cancer. Further research into these pathways and their interactions may provide new insights into the mechanisms underlying PNI and potential targets for therapeutic intervention.

### 3.3 Association of PNI and immune cell infiltration in OC

The nervous system exerts an influence on the tumor microenvironment through the immune-modulating effects of neurotransmitters. Tumor microenvironment (TME) encompasses a complex ecosystem of cells, molecules, and other factors that surround and interact with cancer cells. These neurotransmitters can either promote or inhibit tumor inflammation and immune responses, thereby impacting the efficacy of immunotherapy. Due to the strong association between immune cell infiltration, cancer progression, and clinical outcomes, we then explored whether the status of PNI in TME immune cell infiltration played a role in the process (Karakasheva et al., 2018; Cervantes-Villagrana et al., 2020). An evaluation of 24 TME cell infiltrations in PNI-positive and PNI-negative OC samples was conducted. According to the immune infiltration analysis, immunocytic infiltration was significantly different in PNI-positive *versus* PNI-negative OC samples. It is noteworthy that PNI positivity was negatively correlated with inflammation by innate immune cells in OC neoplastic tissue, including macrophages, natural killer cells and monocytes (Figure 2F). Our work also examined the somatic mutation landscapes of tumors under the different statuses of PNI using TCGA-OC cohort data. There were significant differences in the frequency of TP53 mutations between PNI-positive and PNI-negative OC samples: 100% in PNI-positive OC samples, and 77% in PNI-negative OC samples (Figures 2G,H).

### 3.4 Bitter taste sensing type 2 receptors (TAS2Rs) and perineural invasion in OC

**FIGURE 3:**
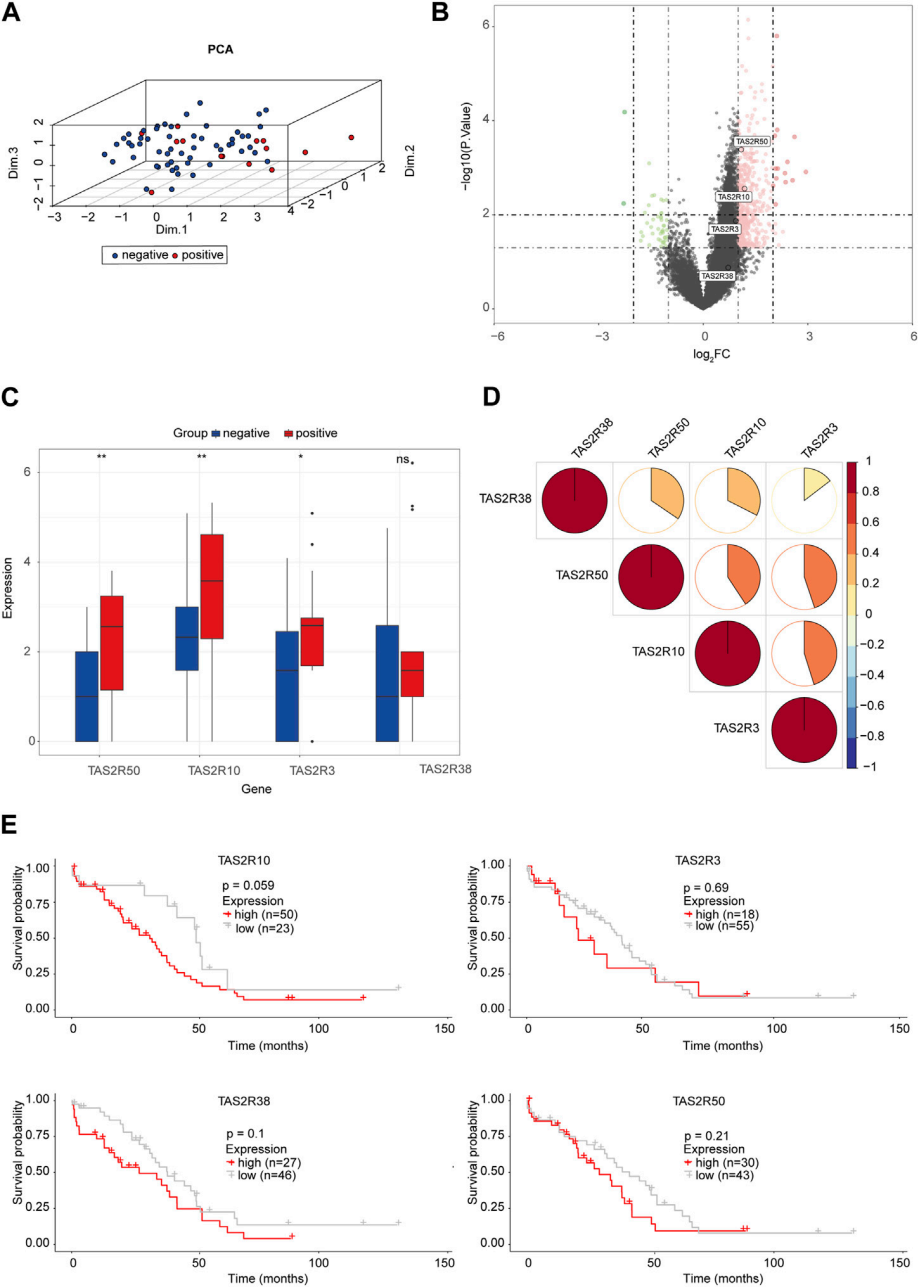
The Bitter Taste Receptors (TAS2Rs) Family Proteins and Perineural Invasion in OC **(A)** Principal component analysis for TAS2R3, TAS2R10, TAS2R50 revealed two disjoint populations. **(B)** Volcanograms built with *p* < 0.05 and |logFC|≥1 as cutting criteria. Red, upregulated genes; Green, downregulated genes. The circle represented each gene and the identified TAS2R3, TAS2R10, and TAS2R50. **(C)** TAS2R3, TAS2R10, and TAS2R50 expressed in the PNI-positive and PNI-negative OC tissues (*p < 0.05; **p < 0.01; ***p < 0.001). **(D)** Correlations among TAS2R3, TAS2R10 and TAS2R50 were analyzed by spearman analysis. Negative associations were labeled in blue and positive associations were labeled in red. **(E)** Survival analyses for TAS2R3, TAS2R10, and TAS2R50 in OC with different PNI statues.

Taste molecules are considered to be the G protein-coupled receptor (GPCRs) family, the bitter taste sensing type 2 receptors proteins (Seo et al., 2017). It has been reported that humans have about 25 functional TAS2R genes (Grassin-Delyle et al., 2019). Originally, it was believed that TAS2Rs, whose ligands are bitter substances, expressed themselves only in the mouth. It has been shown that TAS2Rs are expressed extra-orally as well, suggesting that they could be promising drug targets (Gaida et al., 2016). Of note, we found TAS2R10, TAS2R3, and TAS2R50 were highly expressed in PNI-positive OC samples (Figure 3B). TAS2Rs may have significant relationships with the status of PNI. We investigated the relationship between those molecules at protein levels (Figure 3D). A significant increase in TAS2R10, TAS2R3, and TAS2R50 expression was observed in PNI-positive tumor tissue samples compared to PNI-negative (Figure 3C). PCA was used to reduce the dimensionality of the data and identify whether those molecules could distinguish the status of PNI in OC samples. We found that PNI-positive tumor samples were disjoint from PNI-negative, indicating there was a significant difference in expression patterns of those proteins between PNI-positive samples and PNI-negative tumors (Figure 3A). Notably, we found high expression of TAS2R3, TAS2R10, and TAS2R58 correlated with poor prognosis in 73 samples (Figure 3E).

**FIGURE 4:**
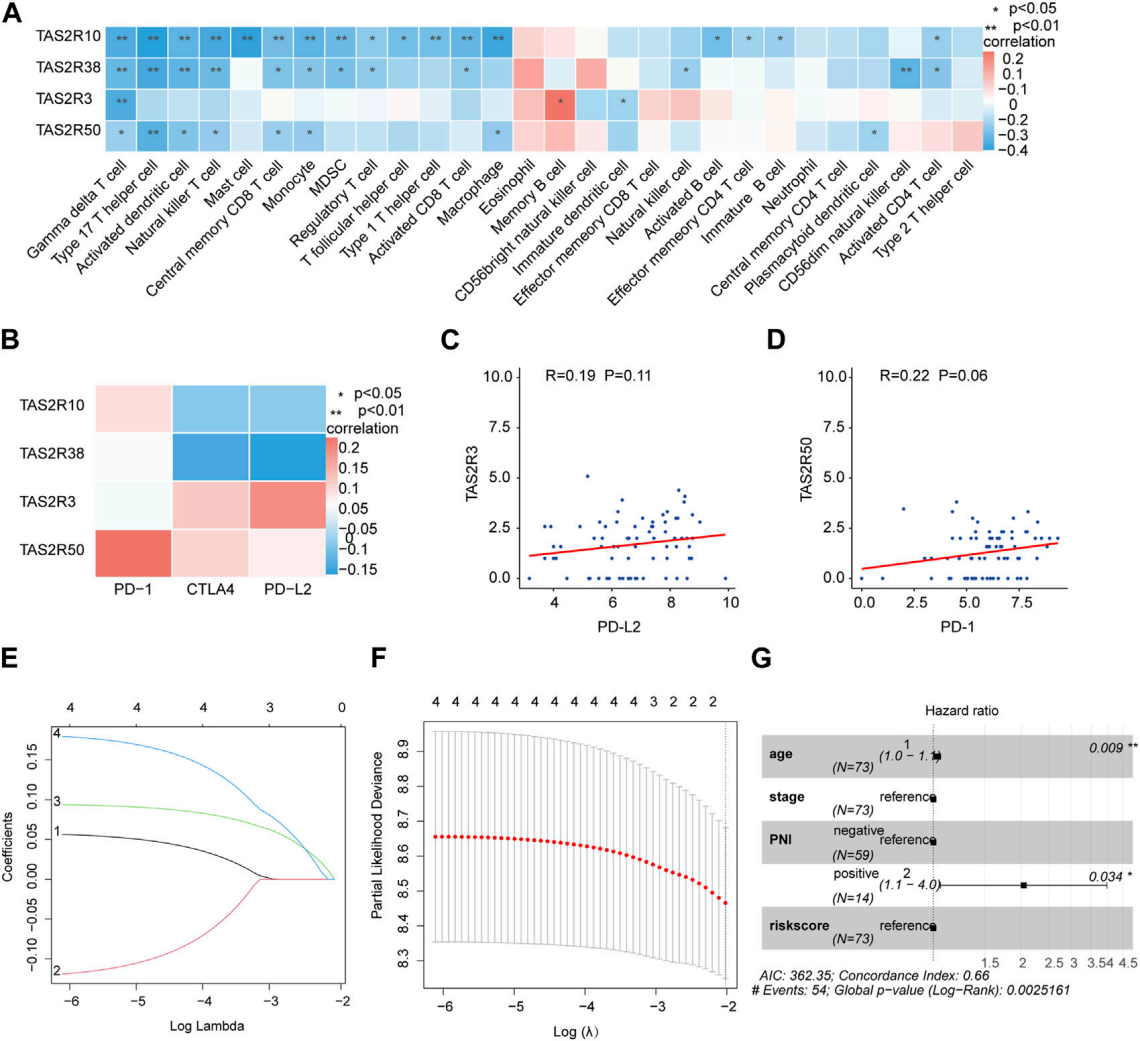
Construction of prognostic and immunotherapeutic relevant gene signatures based on TAS2R3, TAS2R10, and TAS2R50. **(A)** Association among TAS2R3, TAS2R10, TAS2R50 and various TME-infiltrating cells categories. Red colored as positive; blue colored as negative. **(B)** The correlation between TAS2R3, TAS2R10, TAS2R50 and immune checkpoint molecules. **(C)** Association of TAS2R3 and PD-L2 expression. **(D)** Correlates of TAS2R50 expression with PD-1 expression. **(E)** Least absolute shrinkage and selection operator factors curves for TAS2R3, TAS2R10 and TAS2R50. **(F)** Adjustment of parametric choices by 10-fold crossing verification within the LASSO modelling. **(G)** The forest diagram reveals that riskScore by using multivariate analysis was a stand-alone biological indicator of prognosis.

We quantified by ssGSEA analysis the infiltration of the immune cells and their immunological functions to assess the potential relations of key molecules (TAS2R3, TAS2R10 and TAS2R50) with immune-related features. According to the Spearman correlation test, protein levels of the key molecules and the degree of immune infiltration were negatively correlated (Figure 4A). There was a strong association between immune escape and immune checkpoint molecule expression. Furthermore, in our study, we investigated the expression of immune checkpoint molecules, including PD-1, PD-L2, and CTLA4, and found a positive correlation between the expression of TAS2R3 and TAS2R50 and the expression of these immune checkpoint molecules, as shown in Figures 4B–D. These findings suggest a potential link between the nervous system and the immune system within the tumor microenvironment, which could have implications for the response to immunotherapy in ovarian cancer patients with PNI positivity. This observation represents an important step towards understanding the complex interactions between the nervous system, immune system, and cancer cells, and may facilitate the development of more effective immunotherapeutic strategies for ovarian cancer patients with PNI positivity. To better predict the outcome of patients with different statuses of PNI in OC, we conducted LASSO Cox regression on the key genes. An average expression of representative marker genes was used to determine the related score (Figures 4E,F). Independent multi-variate prognosis analyses showed that risk score may be an independent prognostic factor in the prediction of survival in OC. (Figure 4G).

### 3.5 The Bitter Taste Receptors (TAS2Rs) Family Proteins expression in the cancerous perineural invasion

**FIGURE 5:**
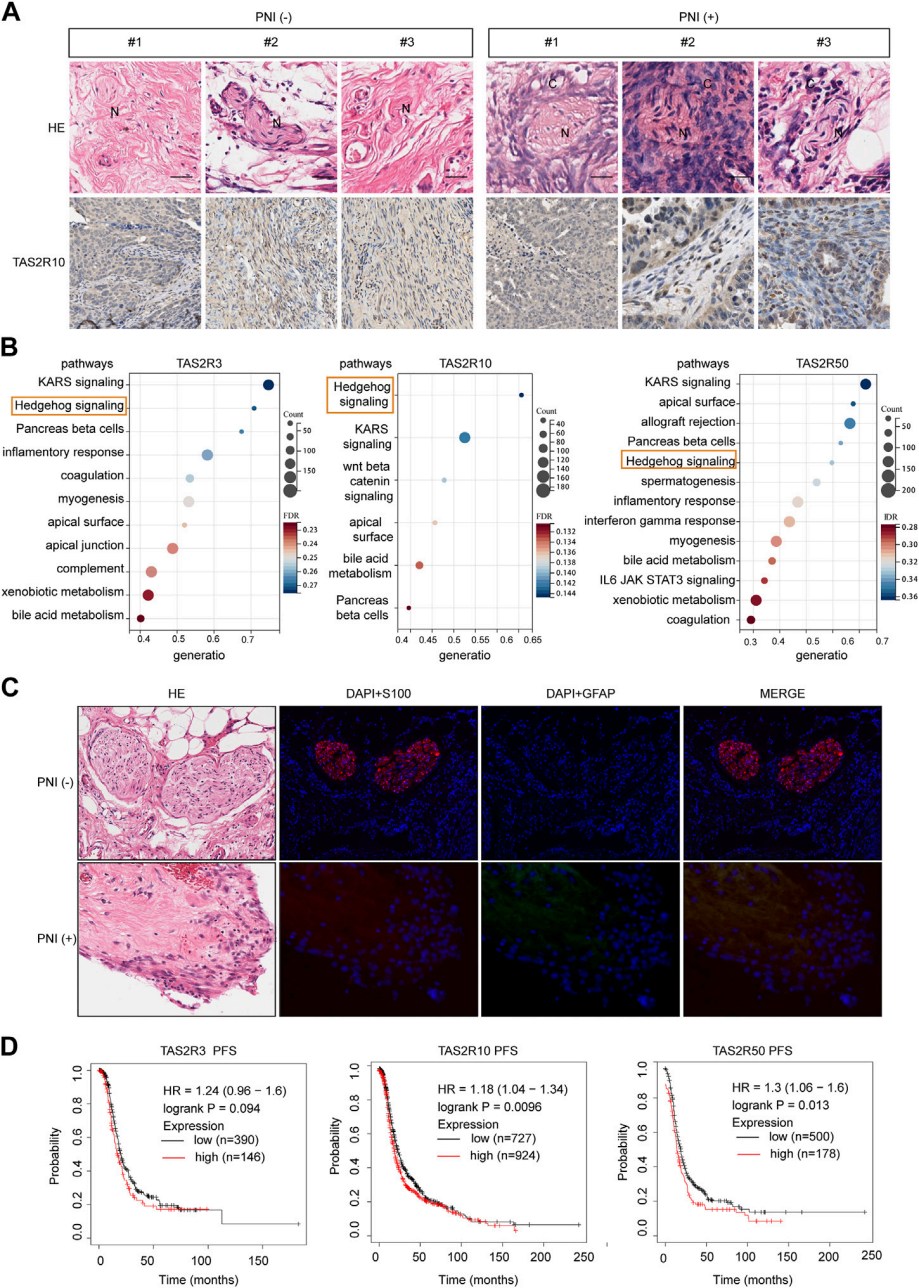
The Bitter Taste Receptors (TAS2Rs) Family Proteins expression in the cancerous perineural invasion. **(A)** Representative HE images and corresponding immunohistochemical (IHC) images of TAS2R10 protein expression in ovarian cancer (OC) samples from the PNI-positive and PNI-negative groups are shown. Scale bar: 100μm; 50 μm. **(B)** KEGG enrichment of differentially expressed genes induced by TAS2R3, TAS2R10, TAS2R50, and TAS2R38. The hedgehog signaling pathway was marked. **(C)** GFAP (green) and S100 (red) colored nerve slides of OC patients with representative pictures and respective H&E sections. Contrast images were taken from the same patient with different tissue sections from cancer-free ovarian areas. Scaling bars represent 20 μm. **(D)** A Kaplan-Meier assay of OC based patients with TAS2R3, TAS2R10 and TAS2R50 expressing in the TCGA cohort.

TAS2R10 expression pattern in human OC tissues was investigated for their role in PNI development and progression using 100 PNI-positive or PNI-negative clinical samples. H&E staining was used to distinguish OC cells and nerves. Immunostaining of these specimens revealed that PNI-positive samples expressed higher level of TAS2R10 than PNI-negative samples (Figure 5A). Further exploration of the mechanisms underlying TAS2Rs on PNI in OC was carried out via KEGG enrichment analysis, which revealed that the same pathways were enriched in TAS2R10, TAS2R3, and TAS2R50, such as upregulation of Hedgehog signaling. Previous studies have shown that Hedgehog ligands derived from muscle sensory nerves drive signaling in basal cell carcinoma (Hanahan et al., 2023). Recent studies showed that Schwann cells induce cancer invasion through direct contact (Hirth Gandla et al., 2020; Zhang et al., 2022). After PNI, Schwann cells in the distal nerve begin to dedifferentiate, a process reliant on the ubiquitin-proteasome system (Tian et al., 2022). We also wanted to know whether PNI occurs in OC through the Schwann cell dedifferentiation pathway. Our first step was to determine whether PNI induced Schwann cell dedifferentiate by testing the level of GFAP protein. Anti-GFAP and anti-S100 antibodies were used to stain sections of tumor tissue with and without PNI (Deborde et al., 2016; Fukuda et al., 2022). As compared with PNI-negative samples, nerves showed higher GFAP levels in PNI-positive samples (Figure 5C). Our study revealed that high expression of TAS2R10, TAS2R3, and TAS2R50 is significantly associated with poor prognosis in ovarian cancer, as demonstrated by our analysis of clinical samples (Figure 5D). These findings suggest that these receptors may serve as potential prognostic markers for ovarian cancer, and further research is needed to fully understand their role in disease progression and to determine whether they could be targeted for therapeutic purposes.

## 4 Discussion

A neoplastic microenvironment is characterized by multidirectional communication between nerves and cancer cells (Zahalka et al., 2020). During PNI, tumor cells and peripheral nerves may interact reciprocally, with the invading tumor possibly responding to pro-invasive signals from the peripheral nerve environment (Sejda et al., 2020). The molecular mechanisms involved in PNI and OC have been studied in relatively few studies, which suggests that the phenomenon need further study. In order to prevent and treat tumors effectively, we must understand and elucidate the molecular basis of PNI in tumors and find new targets for treatment. Our study utilized multi-omics data and clinical data from TCGA OC types to uncover global variations in perineural invasion regulator genes at the genetic, epigenetic, and transcriptional levels. The findings contribute to a greater understanding of the molecular events underlying PNI, as well as elucidating the cancer cell requirements that are necessary for this process. In short, we observed that PNI was an influential independent risk factor for the low overall survival of OC patients. Moreover, the Bitter Taste Receptors (TAS2Rs) Family Proteins may influence tumor progression by regulating PNI.

It has been reported that PNIs strongly correlate with aggressive tumor behavior, disease recurrence, and reduced survival rates in many cancers (Jiang et al., 2022). During PNI, tumor cells and nerves have reciprocal signaling interactions, with the invading tumor possibly acquiring the ability to respond to pro-invasive signals within the peripheral nerve milieu (Liebig et al., 2009). In spite of the fact that PNI is poorly understood and its mechanisms remain unclear. One of the most important mechanisms was that neurotransmitters released by nerves infiltrate the tumor microenvironment and stimulate cancer cell survival, proliferation, and spread; in turn, cancer cells promote nerve growth (Yin et al., 2021). A high level of expression of neurotrophic factors including BDNF and NGF correlates closely and positively with a high FIGO stage, lymph node metastasis, the growth of neurons, and poor long-term outcomes in OC (Retamales-Ortega et al., 2017). As reported, intratumoral NE exerts a neurotrophic effect through the induction of BDNF, whose high expression related to adverse outcomes of OC patients. Therefore, the elimination of tumor innervation may provide a new idea for the treatment of OC.

It is important to understand that the tumor microenvironment, including tumor cells, immune cells, extracellular matrix and mesenchymal tissue, acts in the tumor and PNI development. (Jiang et al., 2022). There were two distinct phenotypes of macrophages: a classical, pro-inflammatory phenotype known as M1 and an alternative, anti-inflammatory phenotype known as M2 (Meinel et al., 2011; Anderson et al., 2021). There was a study that demonstrated NE can combine with beta2-adrenergic receptors attached to macrophages directly and differentiate macrophages toward the anti-inflammatory M2 phenotype to promote the growth of OC. In our study, the immune cell infiltration was significantly different among PNI-positive *versus* PNI-negative OC samples. It Is noteworthy that PNI positivity has a negative correlation with the infiltration of innate immune cells in OC tumor tissues, including macrophages, natural killer cells, and monocyte. Direct causal links were difficult to establish between immune cells and PNI, but there is a certain relationship between them that was undeniable. Consequently, it is considered a new clue for OC immunotherapy.

The most lethal gynecological malignancy in women is OC, which accounts for one-third of all cancers in women (Torre et al., 2018). There has been reported that bitter taste receptors were highly expressed in OC(Jeruzal-Swiatecka et al., 2020). Not only that TAS2R16 induced neurite outgrowth in the human neuroblastoma cells via the ERK pathway (Jeruzal-Swiatecka et al., 2020). Furthermore, TAS2R38 played an important role in promoting OC tumorigenesis and was therefore a potential therapeutic target (Gaida et al., 2016). According to public databases and our clinical samples, we found TAS2R10, TAS2R3, and TAS2R50 were highly expressed in PNI-positive OC samples. Notably, high expression of TAS2R3, TAS2R10, and TAS2R58 was strongly correlated with poor prognosis in OC samples. This means that TAS2Rs expression may still serve as a useful biomarker in OC with different PNI statuses. The hedgehog pathway was reported to have a significant role in pancreatic cancer invasion of the perineurium by activating pancreatic cancer cells to secrete high levels of PNI-related molecules (Li et al., 2014). In addition, *in Vitro* and *in Vivo* models of nerve invasion confirmed that abolition of the hedgehog pathway inhibited migration of pancreatic cancer cells to nerves and invasion of the sciatic nerve into the spinal cord. (Li and Wang, 2014). We revealed that the same pathway of Hedgehog signaling pathway was activated in high levels of TAS2R10, TAS2R3, and TAS2R50. As a result, we hypothesized that TAS2Rs might function via the Hedgehog signaling pathway in the progress of PNI.

This is the first study to describe the genomic, transcriptomic, and immunological spectrum analyses of PNI molecules in OC. Consequently, we found that PNI was a powerful predictor of poor prognosis in OC and analyzed its expression pattern, providing new clues for guiding clinical prevention and treatment of OC.

## Data Availability

The datasets presented in this study can be found in online repositories. The names of the repository/repositories and accession number(s) can be found in the article/Supplementary Material.
