# Supplementary material for: Transcriptome analyses reveal new insights on key determinants of perineural invasion in high-grade serous ovarian cancer
Source: Front Cell Dev Biol. 2023 Sep 20;11:1109710. doi: 10.3389/fcell.2023.1109710 (PMC10548129; doi:10.3389/fcell.2023.1109710)
Supplement: Supplementary file 1 [file Table1.docx]

Supplementary Table 1. Gene expression markers for nerve cells

| Neurons type | Markers | Upregulated genes |
| --- | --- | --- |
| Choroid plexus cells | KL, CHMP1A, SLC26A11, SLC23A2, WFIKKN2, SLC2A12, CLDN1, PRLR, SLC29A4, SLC13A4, CLDN2, SLC31A1, SLC4A5, SLC4A2, SLC16A2, HTR2C, SLC4A10, CLIC6, SOSTDC1, TTR, CAR12,  AQP1 | HTR2C |
| Adrenergic neurons | PNMT, DDC, DBH, SLC18A2, NPFF, SLC12A7, SYT1, TH |  |
