# Supplementary figures and images for: Transcriptome analyses reveal new insights on key determinants of perineural invasion in high-grade serous ovarian cancer
Source: Front Cell Dev Biol. 2023 Sep 20;11:1109710. doi: 10.3389/fcell.2023.1109710 (PMC10548129; doi:10.3389/fcell.2023.1109710)

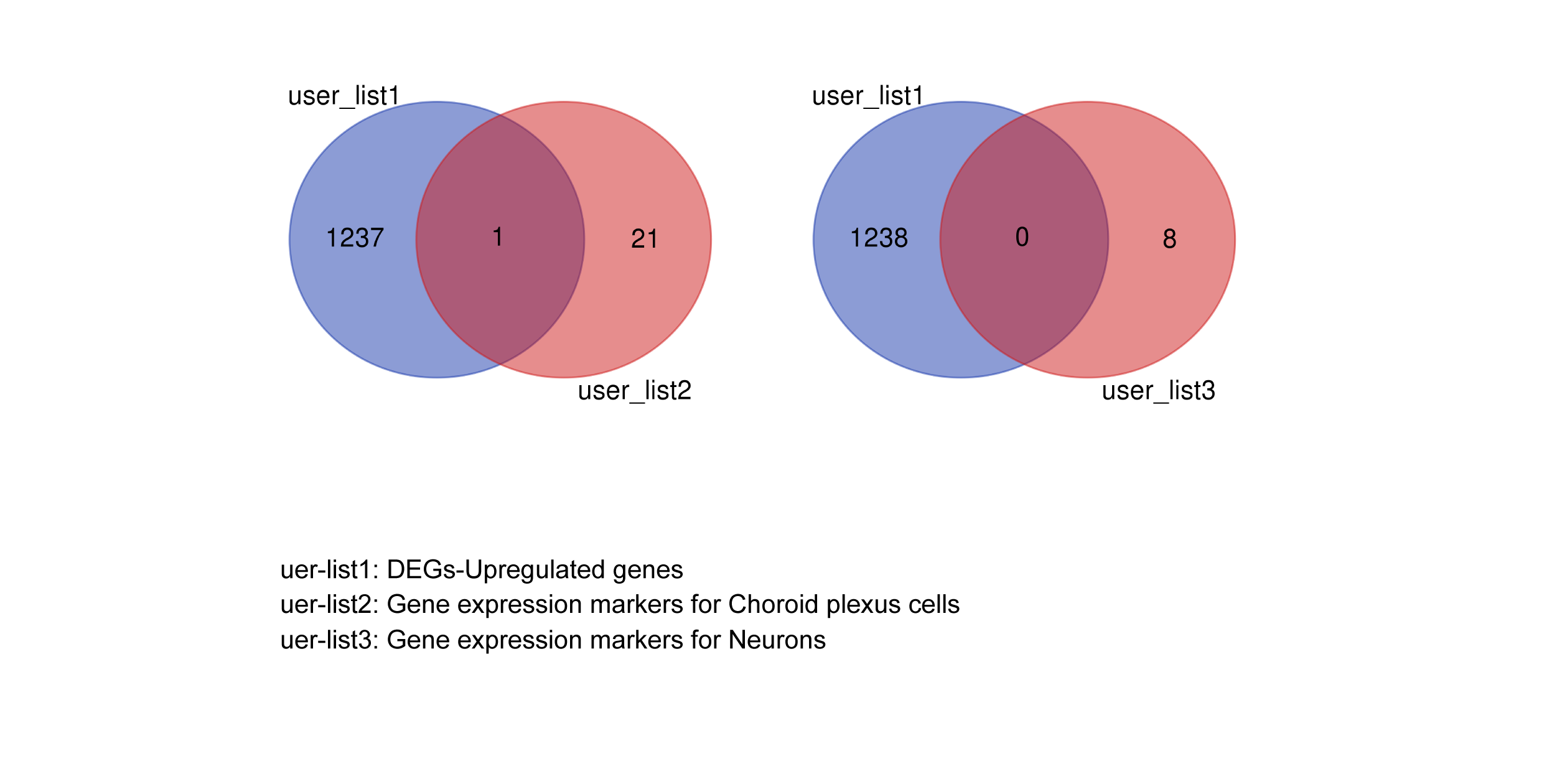

Supplement: Supplementary file 2 [file Image2.TIF]

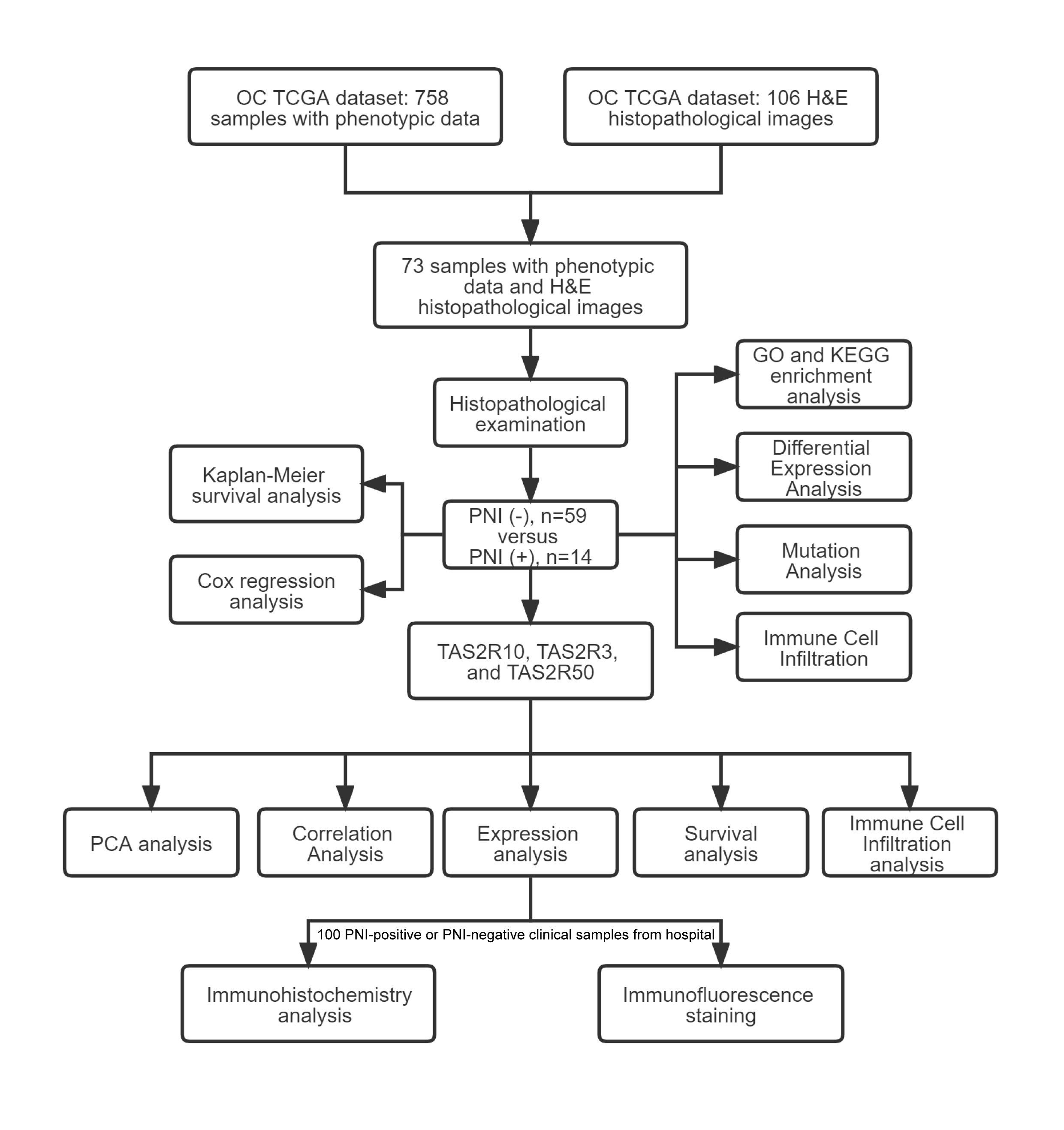

Supplement: Supplementary file 3 [file Image1.TIF]
